# A case study of the reproducibility of transcriptional reporter cell-based RNAi screens in *Drosophila*

**DOI:** 10.1186/gb-2007-8-9-r203

**Published:** 2007-09-28

**Authors:** Ramanuj DasGupta, Kent Nybakken, Matthew Booker, Bernard Mathey-Prevot, Foster Gonsalves, Binita Changkakoty, Norbert Perrimon

**Affiliations:** 1 New York University School of Medicine/Cancer Institute, Department of Pharmacology, First Avenue, New York, NY 10016, USA; 2 Boston Biomedical Research Institute, 64 Grove Street, Watertown, MA, 02472, USA; 3 Department of Genetics, Harvard Medical School, Avenue Louis Pasteur, Boston, MA 02115, USA; 4 Howard Hughes Medical Institute, Harvard Medical School, Avenue Louis Pasteur, Boston, MA 02115, USA

## Abstract

Off-target effects have been demonstrated to be a major source of false-positives in RNA interference (RNAi) high-throughput screens. In this study, we re-assess the previously published transcriptional reporter-based whole-genome RNAi screens for the Wingless and Hedgehog signaling pathways using second generation double-stranded RNA libraries. Furthermore, we investigate other factors that may influence the outcome of such screens, including cell-type specificity, robustness of reporters, and assay normalization, which determine the efficacy of RNAi-knockdown of target genes.

## Abstract 2

A second generation dsRNA library was used to re-assess factors that influence the outcome of transcriptional reporter-based whole-genome RNAi screens for the Wnt/Wingless (wg) and Hedgehog (hh)-signaling pathways.

## Background

In the past few years many groups have successfully conducted high-throughput RNA interference (RNAi) screens using cell-based assays, both in *Drosophila *and mammalian cells, to investigate a variety of biological questions [1-9]. In *Drosophila*, the methodology relies upon the use of long double-stranded RNAs (dsRNAs) which, following uptake by the cells, are processed by Dicer2 into a pool of 21-23 bp small interfering RNAs (siRNAs) [10,11]. These siRNAs silence endogenous gene expression by triggering the cleavage of target mRNAs. In contrast to *Drosophila*, where long dsRNAs of more than 100 bp are used as RNAi reagents, 21-23 bp siRNAs are used directly in mammalian cells to avoid the detrimental interferon response triggered by the cells in response to long dsRNAs [12-15].

The development and application of genome-wide RNAi screens has occurred in parallel with a rapidly evolving understanding of the mechanism of RNAi, including the regulation and processing of dsRNAs, the factors that influence siRNA specificity and efficacy, as well as the biogenesis, expression and function of microRNAs (miRNAs) in cells [10,16,17]. These recent developments have led to a much greater understanding of siRNAs and dsRNAs as RNAi reagents, especially with regards to their specificity in degrading the intended target gene [18,19].

The discovery of 'off-target effects' (OTEs) has played a critical role in promoting a much greater appreciation of various rules dictating siRNA specificity. OTEs were initially recognized as an important source of false positives in mammalian studies using single siRNAs for the knockdown of target genes [13,20]. Subsequently, studies conducted with pools of siRNAs targeting the same transcript revealed that OTEs could be reduced (albeit not always eliminated), as undesirable effects of single siRNAs bearing perfect or partial homologies to other gene coding regions or their 3' untranslated regions were diluted by the pooling method [21-24]. The protection against OTEs provided by pools of siRNAs was the main reason for arguing that OTEs would not be a significant issue in *Drosophila *or *Caenorhabditis elegans *screens, despite the fact that Dicer (RNase III ribonuclease)-mediated cleavage of long dsRNAs could give rise to siRNAs with partial (typically 19-21 bp) sequence complementarity to transcripts other than the intended target. Moreover, the failure to detect the existence of any member of the ubiquitous family of RNA-dependent RNA polymerase (RdRp) in *Drosophila *potentially eliminated the chances of any amplification step of target RNAs, hence limiting the effect of OTEs [25]. As such, OTEs arising from the knockdown of unintended target genes were not thought to be a significant source of cellular phenotypes, and thus were thought unlikely to contribute to the rate of false positives in any high-throughput screen (HTS) in these organisms.

This line of reasoning, however, had not been rigorously tested experimentally and was questioned in a review article by Echeverri and Perrimon [19]. Shortly thereafter, two groups independently reported evidence for OTEs in *Drosophila *RNAi screens [18,19,26,27]. Together, these studies implicated identity stretches as short as 13 nucleotides (nt) for low complexity trinucleotide repeats (for example, CAN repeats) [27] or slightly longer (17-19 nt and greater) for more complex sequence homologies [26] as contributing to false positives in *Drosophila *RNAi screens. Although sequence homology can lead to OTEs, the mere presence of predicted-sequence homology to multiple transcripts does not necessarily translate into OTEs. For example the Kulkarni *et al*. study revealed that 50 of 135 predicted 19 nt off-target sequences (OTs) in a dsRNA designed to target the *PP2A-B*' gene did not cause any changes in expression levels of the corresponding mRNAs. This may reflect the fact that the problematic siRNAs were not produced *in vivo *because of the processivity exhibited by Dicer when acting on dsRNAs [10,17,28,29], or if they were, that they were not effective in knocking down their cognate targets. Thus, *in silico *prediction of OTs will almost always over-estimate the incidence of OTEs that might occur with dsRNAs in an experimental setting.

Here we investigate the extent to which OTEs contribute to the rate of false positives in the Wnt/Wingless (Wg) and Hedgehog (Hh) transcriptional reporter based RNAi-screens that were conducted in our laboratory [3,6]. These screens were performed using a first generation library of dsRNAs [2], referred to as DRSC1.0, which was assembled prior to recognition of the OTE issue. To avoid the issue of sequence-specific OTEs in genome-wide screens, we generated the DRSC 2.0 screening collection, and assembled as well an independent collection, DRSC-validation (DRSC-v), for independent confirmation of hits identified in initial screens. These libraries are composed of dsRNAs largely free of any predicted OTs. We used dsRNAs from the DRSC-v collection to target candidate genes obtained as 'hits' in our previous Wg and Hh screens. Our data show that the activity of 73% and 51% of the DRSC1.0 dsRNAs affecting the Wg- and Hh-responsive transcriptional reporter read-outs, respectively, could be reproduced in assays using the new validation dsRNAs. While cross-reacting sequences in dsRNAs can clearly lead to an increase in false positives, we also describe how other factors, such as cell-type specificity, use of specific normalization vectors, and properties of the transcriptional reporters, can have a major impact on the outcome of reporter-based RNAi screens.

## Results and discussion

### New generation of DRSC dsRNA libraries

The overriding conclusion from previous studies was that the best way to provide better accuracy in RNAi screening with long dsRNAs was to design the dsRNAs as specifically as possible and use more than one dsRNA to rule out false positives [18,19,26] (Supplementary Figure 2 in [26]). To achieve this goal, we assembled a new dsRNA collection in which all (7,692) dsRNAs from the initial DRSC1.0 collection predicted to cross-hybridize with unintended targets were replaced with new, independently synthesized dsRNAs free of OTs. These new dsRNAs were combined with the rest of the original dsRNAs that target only the intended genes to make the 'DRSC 2.0' collection. For each new dsRNA, we selected a region that: was shared by all isoforms (if more than one transcript was transcribed from that gene); and was devoid of any predicted 19 nt sequence identity to other genes. Unique primers flanked with the T7 promoter were designed and used to amplify fly genomic DNA. Each PCR product was purified and an aliquot was transcribed *in vitro *to yield the corresponding dsRNA. In addition, approximately 6,000 dsRNAs in the original DRSC1.0 collection targeted genes solely predicted computationally (the so-called Heidelberg dsRNAs [2,30]. We removed the vast majority of these dsRNAs from the DRSC2.0 collection as there was little evidence to support the idea that their targets corresponded to *bona fide *genes, and kept only those (about 10%) for which there was independent confirmation of expression or of the validity of gene prediction in subsequent releases of the *Drosophila *genome annotation [31].

**Figure 2:**
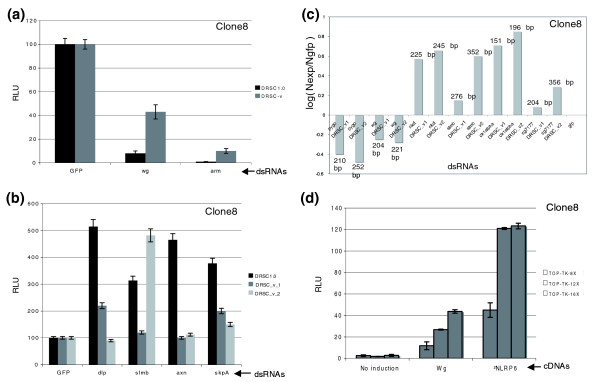
Properties of dsRNAs and reporter genes can influence the sensitivity of the RNAi assay. **(a-c) **The dynamic range of validation dsRNAs is smaller than that of the DRSC1.0 dsRNAs, which could potentially increase the rate of false negatives. The effects of dsRNA-mediated knockdown of known Wg-pathway regulators were tested by measuring their effect on the Wg reporter activity. DRSC1.0 and DRSC-v dsRNAs were compared in parallel. Knockdown of *wg *and *arm *using DRSC1.0 dsRNAs resulted in 90% and 99% reduction in Wg-reporter activity, respectively ((a), black bars). On the other hand, validation dsRNAs for *wg *and *arm *reduced reporter activity by only 58% and 90%, respectively ((a), grey bars), suggesting that the DRSC-v dsRNAs for some genes may not be as efficient in targeting their endogenous transcripts. Some of the validation dsRNAs ((b), grey and light grey bars) targeting known negative regulators did not produce robust effects on reporter activity compared to their DRSC1.0 counterparts ((b), black bars), including *dlp*, *axn*, *skpA *and one dsRNA in the case of *slmb*. Two independent validation dsRNAs targeting the same gene could influence reporter activity to different extents (compare DRSC_v1 and DRSC-v2 dsRNAs for each target gene in (c)). **(d) **Finally, the number of Tcf binding sites in the Wg responsive luciferase reporter gene can affect the robustness (fold change) upon induction by Wg. Reporter gene carrying 8 (white bar), 12 (grey bar) or 16 (black bar) sites were co-transfected with wg expressing cDNA. Increasing the number of Tcf binding sites increased the fold induction of the luciferase reporter upon addition of both Wg or ΔNLrp6 to induce the Wg pathway. All luciferase reporter assays were performed in 4 replicas and error bars represent the standard error between the four data points.

Furthermore, to confirm the effects of dsRNAs identified in the initial screens, we decided to generate DRSC-v, which is composed of a set of second or third independent dsRNAs targeting a gene identified in a screen, even if the original dsRNA had no predicted OTs. To date, this ever expanding library contains about 7,000 distinct dsRNAs targeting 4,100 genes. The major consideration that went into the design of the validation dsRNAs was that, other than being free of predicted OTs, they should, if possible, not overlap with any of the dsRNAs used in the original DRSC1.0 collection. This was necessary to fulfill the requirement that a set of completely independent dsRNAs be used to confirm the original findings in a primary screen. However, because of the design restrictions, the regions of each gene that were available for targeting were much smaller than what was used for the original DRSC1.0 set. As a result, a majority of the validation dsRNAs are about 200-300 bp in length, as opposed to an average size of 400-500 bp for dsRNAs in the DRSC1.0 screening collection. Although we have failed to observe a strong correlation between size and efficacy in experiments reported here, it remains to be determined whether the smaller size of the validation dsRNAs might lead in some cases to lesser efficiency in knock-down as the probability of generating efficient siRNAs *in vivo *might be proportional to length.

### Re-screening candidate dsRNAs isolated in the screen for regulators of the Wg and Hh pathway with OT-free dsRNAs

**Figure 1:**
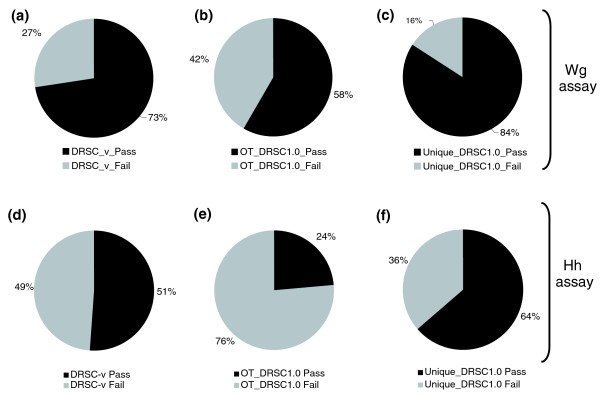
Re-screening the candidate genes identified in previous Wg and Hh screens using the DRSC-v dsRNAs. **(a-c) **Wg assay: 148 of 204 (73%) dsRNAs screened had reproducible effects on the Wg responsive reporter activity (a); 58% of the DRSC1.0 dsRNAs that were predicted to have OTEs repeated in the validation screen (b); while 84% of DRSC1.0 dsRNAs that were predicted to have ≥5 OTs could be reproduced with DRSC-v dsRNAs (c). **(d-f) **Hh assay: 179 of 351 (51%) validation dsRNAs had reproducible effects on the Hh responsive reporter activity (d); 24% of the DRSC1.0 dsRNAs that were predicted to have OTEs repeated in the validation screen (e); while 64% of DRSC1.0 dsRNAs that were predicted to have ≥5 OTs could be reproduced with DRSC-v dsRNAs (f).

To examine the issue of false positives in previously published screens for the Wg and Hh signaling pathways [3,6], we have re-assessed the effect of knocking down candidate genes using the new validation dsRNAs (Figure 1). We used the same dual luciferase-reporter assays as previously reported [3,6]. Since the efficacy of target knockdown could depend on the specific region of a given gene towards which a given dsRNA is designed, we used two independent OT-free dsRNAs for most candidate genes identified in the original screens.

#### Wnt/Wg re-screen

The *Drosophila*-optimized dTF12 and mammalian-cell optimized STF16 reporters were used for validation screening of the candidate Wg-regulators. In this analysis, we re-screened only 204 of 238 dsRNAs that were previously reported in the Wg screen. The 34 dsRNAs that were omitted from the validation screen were those that targeted the *in silico *predicted (Heidelberg annotated) genes [2,30]. For 73% (148 of 204) of the genes isolated in the original Wg screen, at least one new DRSC-v dsRNA showed similar effects on the activity of the Wnt/Wg-responsive luciferase reporter as the original dsRNA (Figure 1a; Additional data files 1&4). In addition, for approximately 40% (80 of the 204) of the original candidate genes tested, two independent OT-free dsRNAs had the same effect on the Wg reporter assay as the original dsRNA (Additional data file 1). Thus, while using multiple independent validation dsRNAs are useful to confirm hits, this approach alone is not definitive in confirming hits because in 68 cases (of the 204 genes screened), one out of three dsRNAs tested failed to give consistent results with the other two. This discrepancy most likely reflects that dsRNAs are not equally effective in knocking down target genes, perhaps as a result of differences in properties between original and validation dsRNAs. In our previous Wnt/Wg screen, we had identified 91 dsRNAs that shared greater than 5 possible 19 nt exact overlaps with other genes that could potentially result in non-specific, OT-related effects on Wg signaling activity (as described in Supplementary Figure S1A and Supplementary Table 2 in [3]). Interestingly, 58% (53 of 91) of those candidate dsRNAs were validated in the re-screen using independent dsRNAs that do not share 19 nt homology with other transcribed genes (Figure 1b). On the other hand, of the 113 dsRNAs originally identified as candidate 'hits' in the Wg screen that had ≥5 19 nt OT identities, 85% (95 of 113) repeated using DRSC-v dsRNAs (Additional data file 2). In conclusion, our data suggest that much better reproducibility is observed with dsRNAs that lack any predicted 19 nt sequence overlap with other transcripts.

#### Hh re-screen

The GL3-ptcΔ136 reporter described by Nybakken *et al*. [6] was used for re-screening the candidate genes isolated in the Hh-signaling screen. For the Hh assay, one or two new dsRNAs were generated targeting 351 of the genes found in the original screen (as with the Wg screen, it should be noted that the Heidelberg annotated presumptive genes were left out of the set to which new validation dsRNAs were generated). Of the 351 candidate Hh signaling genes targeted by the DRSC-v dsRNAs, 51% (179) had at least one new dsRNA score as a hit again in the GL3 assay (Figure 1d, Additional data file 3). Of the 351 genes retested, 285 had two, separate dsRNAs in the DRSC-v collection, and 66 had only one DRSV-v dsRNA. Of the 66 genes, 34 (52%) were re-confirmed with the single available DRSC-v dsRNA. Of the 285 genes re-tested with 2 new dsRNAs, 82 (29%) repeated as hits with both validation dsRNAs, while 22% (63) repeated as a hit with 1 one of the 2 validation dsRNAs (Additional data file 3). In the original Hh screen, 39% (197) of the candidate genes had >5 potential OTs when looking at possible 19 nt overlaps with other genes. Of these 197, 110 were re-tested in the DRSC-v screen (Additional data file 3). Only 24% (26 of 110) were found to have at least one new dsRNA that gave a similar effect as the original dsRNA in the GL3 assay (Figure 1e, Additional data file 3). Of the 241 genes that we retested that had ≥5 potential 19 nt OTs in the original screen, 64% (153) were validated using DRSC-v dsRNAs (Figure 1f). Thus, similar to the Wg screen, much better reproducibility was observed in the Hh screen with genes that, in the original screen, had been identified using dsRNAs lacking significant 19 nt sequence identity to other transcripts.

### Analysis of *in silico *prediction of OTs and 'repeat-rate' in Wg and Hh validation screens

Our results suggest that there is not necessarily a strict correlation between the rate of false-positives and dsRNAs with multiple potential OT sequences. For the Wg screen, 58% of the genes isolated in the original screen that had >5 potential OT sequences can be revalidated using multiple, independent OT-free dsRNAs (Figure 1b), while only 24% of the genes found in the Hh screen that had >5 potential OTs could be revalidated using multiple, independent OT-free dsRNAs (Figure 1f). Given a lack of strict correlation between the presence of *in silico *predicted 19 nt homologies and false positives, results obtained with dsRNAs containing sequence homologies to other genes should not be disregarded as artifacts without further testing. Indeed, in the Hh screen, two very strong hits, *combgap *(*cg*), a known regulator of Hh signaling, and *Smrter *(*Smr*), a novel regulator of Hh signaling, were initially identified using dsRNAs with >400 potential 19 nt OTs. Retesting with two validation dsRNAs demonstrated that both are indeed strong regulators of Hh signaling.

Conversely, our data also argue that not all dsRNAs targeting a gene are effective in knocking down that gene, regardless of possible OTEs. This notion is supported by the fact that, in the Wg screen, the use of independent dsRNAs confirmed 84% of DRSC1.0 dsRNAs that were not predicted to harbor any 19 nt homology (Figure 1c). The remaining 16% that could not be confirmed could be due to the fact that certain dsRNAs may not be effective at knocking down their cognate target or that additional contributing features in these dsRNAs (other than the strict 19 nt homology) might cause OTEs. Similarly, in the Hh screen independent dsRNAs confirmed 64% of the DRSC1.0 dsRNAs that were not predicted to harbor any 19 nt homology (Figure 1f). However, it is also important to consider the possibility that for those dsRNAs with no predicted 19 nt OT that failed to repeat with validation dsRNAs, they might in fact have an OT effect at less than 19 nt, perhaps in the 13-18 nt window.

Overall, the validation rate for the entire Wg screen (73%) is similar to the average repeat rate between the >5 19 nt homology containing (58%) and the OT-free candidate dsRNAs (85%) reported in the previous Wg screen using the DRSC1.0 library. Furthermore, it is similar to the validation rates reported in another published screen [4]. Similarly, for the Hh screen, 51% of the candidate dsRNAs could be re-validated using the OT-free validation dsRNAs from the DRSC-v library, a proportion similar to that passing secondary assays using the DRSC1.0 library [6].

### Properties of dsRNAs and luciferase reporters that may affect assay sensitivity

*In vitro *cell culture studies have suggested that the efficacy of knockdown of any given target mRNA is directly proportional to the length of the dsRNA introduced into a cell [17,32]. A longer dsRNA would typically produce a greater number of siRNAs upon Dicer-mediated cleavage and, hence, increase the likelihood that one or more of the siRNAs produced would efficiently knock down the targeted gene. However, in our overall analysis we could not find a statistically significant correlation between size of dsRNAs and magnitude of phenotype and we have clear examples where the converse is true. For example, knockdown of *supernumerary limbs (slmb)*, a known negative regulator of the Wg-pathway, using a shorter validation dsRNA from the DRSC-v collection had a greater effect in increasing reporter activity compared to the original DRSC1.0 dsRNA, suggesting that the difference in length alone could not always explain the reduced efficiency in the generation of a phenotype (Figure 2b, DRSC-v2 dsRNA for *slmb*).

However, in the Wg assay, we do see a rough correlation between dsRNA size and dynamic range. In fact, when we compared the effects of dsRNAs from the DRSC1.0 collection targeting some of the known or newly identified candidate modulators of the Wg signaling pathway with those from the DRSC-v collection, we observed a surprising difference in the dynamic range in the effect of dsRNA knockdown on Wg-luciferase reporter activity (Figure 2). In many cases, it was significantly reduced when DRSC-v dsRNAs were used when compared to the corresponding DRSC1.0 dsRNAs (Figure 2). For example, using dsRNAs directed towards positive effectors for the Wg signaling pathway, we found that knocking down *armadillo *(*arm*) with a DRSC-v dsRNA reduced reporter activity by 90% as opposed to 99% with the DRSC1.0 dsRNA, in spite of transfection with equal amounts (100 ng) of the two dsRNAs (Figure 2a). Similarly, knocking down pathway activity using a DRSC-v *wg *dsRNA reduced reporter activity by approximately 55-60%, which was in sharp contrast to the DRSC1.0 *wg *dsRNA that reduced pathway activity by 90% (Figure 2a). On the other hand, knocking down some of the Wg-specific negative regulators, such as *slmb*, *skpA *and *dally-like protein *(*dlp*), or a novel candidate regulator *CG7177 *resulted in a moderate increase in reporter activity with only one of two DRSC-v dsRNAs. In the case of *axin *(*axn*), neither of the two DRSC-v dsRNAs re-validated in spite of *axn *RNAi having a robust effect on reporter activity with the DRSC1.0 dsRNA (Figure 2b,c).

Although more work needs to be done, one possibility to explain the trend is that what really matters is the chance of generating siRNAs with high specificity and efficacy after processing by Dicer. It would be logical to assume in these cases that having a longer dsRNA will increase the chance of getting a better knockdown efficiency. The specificity and efficiency of targeting could be tested at the molecular level by assessing the microarray profile of cells upon knockdown of target genes using dsRNAs of varying lengths. Taken together, these data imply that the region towards which any given dsRNA is directed is also important and that a larger dsRNA may not necessarily be efficient in knocking down the intended target if its sequence intrinsically leads to the generation of poor siRNAs.

Finally, we also noticed that the Wg-responsive luciferase reporter (dTF12 or STF16) used in the screen for novel interactors of the Wg pathway can be highly sensitive to the number of Tcf multimerized sites cloned into the reporter vector. We tested the activity of STF8, STF12 and STF16 using the dual-glo luciferase assay upon the induction of the pathway by co-transfection of cDNA encoding the *wg *gene in *clone 8 *cells (Figure 2d). We find that increasing the number of multimerized Tcf sites from 8× to 16× significantly increased the activation level of the reporter and, hence, the sensitivity of the assay. Addition of more than 16 Tcf binding sites did not enhance the pathway activity any further (data not shown).

### Importance of proper normalization

An important aspect of any quantitative measurement of a biological phenomenon derived from cell-based assays is the need for normalization to account for experimental variations introduced by non-specific factors affecting assay readout. For example, most transient transfection assays need to be normalized for cell viability and transfection efficiency. Luciferase assay normalization is typically achieved by co-transfection of a control reporter expressing Renilla luciferase (RL) along with the experimental firefly luciferase expressing reporter. Two factors are especially important in the design of the control RL. First, the RL should be driven by a ubiquitously expressed promoter that is inert to the activity of the signaling pathway being analyzed. Second, the control Renilla vector should have activity significantly higher than background so that it is immune to background fluctuations inherent to most assays. The choice of the promoters driving RL thus becomes a matter of utmost importance, especially for large genome-scale RNAi screens, as poor normalization can lead to the introduction of significant artifacts in the screen, skewing data analysis and leading to erroneous conclusions.

**Figure 3:**
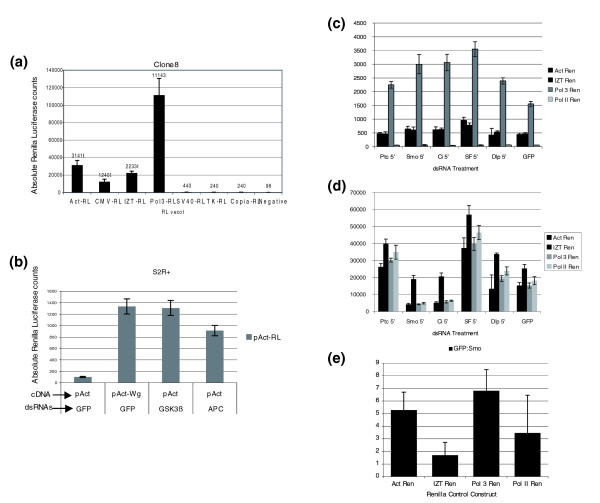
Importance of proper normalization for luciferase assays. **(a) **Assessment of basal activity of the RL vectors that are commonly used in luciferase reporter assays in *Drosophila *clone 8 cells in 96-well plate format. The SV40-RL, TK-RL, and Copia-RL vectors display no activity or very weak basal activity; approximately two to four times above background or negative control (no RL reporter added). CMV-RL displays weak activity (approximately 12 times above background) whereas pIZT-RL and pAct-RL display moderate basal activity (approximately 20 to 30 times above background). PolIII-RL displays the most robust activity among all the RL vectors tested (>1,000 times above background). **(b) **pAct-RL can be activated by transfecting cDNA expressing Wg or by dsRNA-mediated knockdown of known negative regulators (GSK-3β, APC) of the Wg pathway in S2R+ cells, thus rendering it unusable as a control for transfection efficiency and cell viability in luciferase assays. **(c) **RL counts produced by transfection of the indicated Renilla control reporter and treatment with the indicated dsRNA in the Hh signaling assay. **(d) **Firefly luciferase counts produced by the ptcΔ136 reporter when cotransfected with the indicated Renilla control reporter and dsRNA. **(e) **Graph showing the fold difference in ptcΔ136 reporter activity in clone 8 cells treated with *smo *dsRNA versus GFP dsRNA in the presence of the indicated Renilla control reporter. Bars are the ratio of GFP dsRNA treated: *smo *dsRNA treated taken from the data in (c) and (d). All luciferase reporter assays were performed in triplicate and error bars represent the standard error between the three data points.

Control reporters that have been used in various studies include Actin5C-RL (Act-RL), PolIII-RL, pIZT-RL, Copia-RL, TK-RL, SV40-RL and pCMV-RL. We tested the applicability of these vectors in a *Drosophila *RNAi-mediated HTS (in 96-well plates) by measuring their activity in clone 8 cell transient transfections (Figure 3). The transfection protocol and assay conditions were the same as those used in our previously reported Wg and Hh HTSs. As shown in Figure 3a, the raw luciferase values for TK-RL, Copia-RL and SV40-RL either showed no activity or were barely above background. CMV-RL displayed moderate levels of activity. IZT-RL, Act-RL, and PolIII-RL on the other hand displayed robust luciferase activity, although PolIII-RL displayed a three- to five-fold higher activity compared to Act-RL and IZT-RL. Our results suggest that the lack of robust basal activity of TK-RL, Copia-RL or SV40-RL render them unsuitable for HTSs since minute changes in their activity could introduce profound changes in the normalized (N) luciferase activity or 'relative luciferase units' (RLU) as measured by the ratio of firefly and RL (RLU = firefly luciferase/RL). Moreover, the absolute RL counts would not be within the linear range of luciferase activity. PolIII-RL, Act-RL, and IZT-RL, on the other hand, serve as robust control reporters: they have high basal activity and display a broad dynamic range that can accommodate variations in reporter activity due to changes in cell viability, cell proliferation and transfection efficiency. These properties make them well suited for rigorous normalization protocols.

However, for control vectors to be effective tools for normalization of signaling assays, they should not respond to the ligands that induce the activity of signaling pathways. Thus, we tested several control Renilla vectors for their effects on Wg and Hh induction. For Wg activation, PolIII-RL [3,6], pIZT-RL [3], Copia-RL [7], and TK-RL (Promega) did not display any changes in activity upon Wg-stimulation (data not shown). However, the Act-RL vector was strongly activated by Wg induction in S2R+ cells (Figure 3b). To test if the effect on the Act promoter was specific to activation of Wg-signaling, we induced the pathway by dsRNA-mediated knockdown of GSK3β and APC, which are known to be strong negative regulators of the Wg-pathway. RNAi of both GSK3β and APC in S2R+ cells significantly activated Act-RL. Interestingly, scanning the sequence of the Actin5C promoter revealed at least two consensus Tcf binding sites, AaATCAAAG and cGATCAAAG. Whether these sites are true binding sites for Tcf proteins on the Actin promoter needs further testing. However, Act-RL should be avoided for normalization of Wg-induced reporters since its activity is sensitive to the activation of the Wg pathway.

Sensitivity to pathway activation was also tested for RL normalization constructs used in the Hh assay. Hh assays were conducted using the Act-RL [33], PolIII-RL [3,6,33], and IZT-RL [3] normalization constructs. Only PolIII-RL gave RL counts greater than 500, while the Pol II-RL, IZT-RL, and Act-RL constructs all gave less than 500 counts in green fluorescent protein (GFP) dsRNA treated control wells (Figure 3c). As background counts are typically between 50 and 100 in our Hh assays, RL levels for these latter three control constructs did not exceed the threshold of ten times background counts that we feel sufficient to put RL counts in the linear range. Firefly luciferase activity produced by the ptcΔ136 reporter in transfections with the appropriate positive and negative control dsRNAs yielded the expected levels of ptcΔ136 activity when using the PolII-RL, Act-RL, and PolIII-RL control reporters. However, for cells cotransfected with the IZT-RL control reporter, firefly luciferase activity in general is higher for all dsRNA treatments, but is considerably higher than normal in the Smo and Ci dsRNA treated cells (Figure 3d). This is apparently due to transactivation of the ptcΔ136 reporter by the IZT-RL construct itself, thus rendering the IZT-RL unsuitable for use in the Hh signaling assay. Indeed, this can be seen more clearly when the fold differences between GFP dsRNA treated (Hh pathway activated) and Smo dsRNA treated (Hh pathway inactivated) wells are compared. Whereas there is normally a five- to seven-fold difference between these two values in assays in which PolIII-RL or Act-RL vectors are used for normalization, this difference falls to <1.7-fold in assays in which IZT-RL is used as the normalization vector (Figure 3e). While the sensitivity of Act-RL and IZT-RL towards other signaling pathways such as Notch (N) and JAK/STAT and receptor tysosine kinase await further testing, it is imperative that all control RL vectors be subjected to similar tests before using them for normalizing any HTS luciferase-based assays.

### Cell type specificity and robustness of pathway activity for signaling pathways: implications for whole genome RNAi screens

The specificity of proteins regulating the activity of cell signaling pathways is exquisitely regulated in space and time during animal development. Cell type specificity is achieved by the presence of a unique set of proteins and their isoforms, their sub-cellular localization, temporal modulation of their activity, and the quantitative differences in the expression levels of similar sets of factors. Therefore, the choice of a specific cell line in an RNAi HTS screen can result in the identification of different sets of genes in different cell types.

This important issue comes to the forefront especially when comparing similar RNAi screens for the same pathway in two different cell types. For example, three of the dsRNAs (CG6606/l(1)G003, CG5402, CG12993) that were identified as 'candidate hits' in the previously published DasGupta *et al*. [3] screen in clone 8 cells were reported to have no effect on reporter gene activity in S2R+ cells (Supplementary Table S3 in [3]) - an observation independently confirmed by Ma *et al*. [27]. This is a good example of where cell-type specific differences may factor into screen data obtained from two very different cell lines.

**Figure 4:**
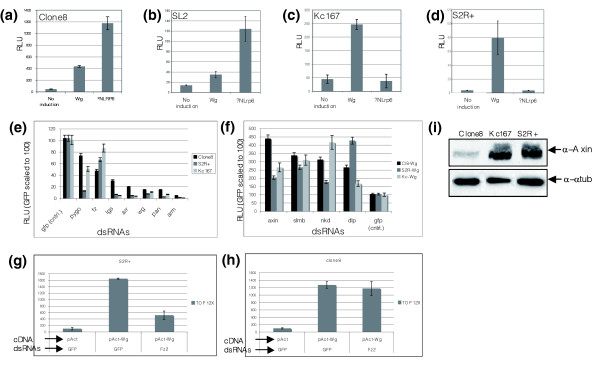
Cell type specificity for signaling pathways. The basal activity and fold induction by Wg or ΔNLrp6 is different in the variety of *Drosophila *cell lines tested. **(a-d) **The *wg *reporter can be induced by the expression of both Wg and ΔNLrp6 in clone 8 and SL2 cells (a,b) but only with Wg in Kc167 and S2R+ cells (c,d). **(e,f) **dsRNA-mediated knockdown of known positive (e) and negative (f) regulators variably affect the activity of the Wg reporter in different cell lines. **(g,h) **Effect of dsRNA-mediated knockdown of *DFz2 *receptor in different cell types. RNAi inhibition of *DFz2 *inhibits Wg pathway activity in S2R+ cells (g) but not in clone 8 cells (h). **(i) **Western blot to detect expression of a negative regulator, Axn in clone 8, Kc167 and S2R+ cell lines. Levels of Axn in clone 8 cells is significantly lower than in S2R+ or Kc167. Anti-α-tubulin antibody was used as a loading control (i). All luciferase reporter assays were performed in 4 replicas and error bars represent the standard error between the four data points.

In order to further address this issue, we investigated the effect of dsRNA-mediated knockdown of known positive and negative regulators of the Wg pathway in a variety of *Drosophila *cells (Figure 4). First, we tested four *Drosophila *cell types, namely clone 8, SL2, S2R+ and Kc167, for their responsiveness towards transfection of *wg *and ΔNLrp6 (a constitutively activated form of the human ortholog of Lrp6 co-receptor; Figure 4). While the Wg-responsive reporter could be activated by co-transfecting cDNAs expressing either *wg *or ΔNLrp6 in clone 8 and SL2 cells (Figure 4a,b), Kc167 and S2R+ responded to Wg only and not to the addition of ΔNLrp6 (Figure 4c,d). This suggests that for a given signaling pathway, there are important differences in the responsiveness of different cell types to different components of the same signal transduction pathway. Moreover, the fold-change in normalized RLUs between Wg-stimulated and Wg-unstimulated was the greatest in the imaginal-disc derived clone 8 epithelial cells, with a 20-25× activation over baseline, followed by S2R+ (10-15× activation), Kc167 and SL2. Thus, for the Wg pathway, different cell types display both qualitative and quantitative differences in their ability to be activated by the Wg pathway.

Additionally, dsRNA-mediated knockdown of several known positive and negative regulators of the Wg pathway showed different effects on modulating pathway activity in the different *Drosophila *cell lines (Figure 4e,f). Whereas downregulation of *pygo *and *legless *(*lgs*) in S2R+ cells had a stronger effect in reducing reporter activity compared to clone 8 or Kc167 cells, RNAi-mediated knockdown of *fz *inhibited reporter activity more efficiently in clone 8 cells than in S2R+ or Kc167 cells (Figure 4e). With respect to the known negative regulators, *axn *knockdown in S2R+ cells did not result in as significant an increase in reporter activity as in clone 8 or Kc167 cells. *Dlp *knockdown, on the other hand, had a greater effect in S2R+ than in clone 8 or Kc167 cells (Figure 4f).

It was particularly interesting to note the failure of robust pathway activation in S2R+ cells by dsRNA-mediated knockdown of *axn *in the light of our observation that ΔNLrp6 fails to activate the Wg-responsive luciferase reporter in S2R+ cells (Figure 4a-d). Recent studies have suggested that constitutively activated ΔNLrp6 or a chimera between the *Frizzled2 *(*Dfz2*) receptor and intracellular cytoplasmic tail of the *Drosophila *ortholog of Lrp6 (encoded by the *arrow *(*arr*) gene) can activate the Wg pathway in a ligand-, GSK-3β-, and *disheveled *(*dsh*)-independent manner [34]. It was also demonstrated that expression of activated Lrp6 could recruit Axn to the plasma membrane and cause its degradation. Taken together, it is possible that the expression levels of *axn *are much higher or that the protein is more stable in S2R+ cells than in clone 8 cells. This could potentially result in an ineffective knockdown of *axn *levels using RNAi, hence explaining the inability of ΔNLrp6 to activate the wg-reporter in S2R+ cells. In order to test this hypothesis, we performed western blot analysis on cellular protein extracts derived from clone 8, Kc167 and S2R+ cells and assessed the expression levels of the Axn protein using anti-Axn antibodies. As shown in Figure 4i, the level of Axn protein is significantly higher in S2R+ and Kc167 cells compared to that in clone 8 cells.

Higher levels of Axn in S2R+ cells could be a result of high levels of Dfz2 expression, which was used as a basis for isolating the S2R+ cell line as a cell-based model for the Wg pathway [35]. Increased Fz2 activity could subsequently activate expression of *axn*, which in mammalian cells, has been shown to be a target of the β-catenin pathway [36,37]. In fact, the basal activity of the *wg *pathway in S2R+ cells is approximately ten-fold greater than in clone 8 cells (data not shown). It is thus tempting to speculate that the presence of the Dfz2 receptor might promote higher basal activity of the pathway in S2R+ and Kc167 cells and more potently activate expression of *axn *compared to that in SL2 or clone 8 cells. This might explain why ΔNLrp6 can efficiently activate the wg reporter in clone 8 and SL2 cells but not in S2R+ or Kc167 cells (Figure 4a-d). In agreement with this notion, dsRNA-mediated knockdown of Dfz2 strongly inhibited the wg responsive luciferase reporter activity in S2R+ cells but not in clone 8 cells, which is most likely why we did not isolate Dfz2 in the previously reported Wg screen (Figure 4g,h; Figure S1 in [3]). Moreover, knockdown of *axn *in clone 8 cells led to a stronger activation of the luciferase reporter compared to S2R+ cells (Figure 4f).

Taken together, the activity of signaling pathways and their modulation by regulatory proteins within the cell can be highly variable in different cell types, depending on the specific cellular context, the quantitative levels of expression of various proteins and their sub-cellular localization. Hence, caution needs to be exercised when comparing the candidate 'hits' obtained in whole-genome screens for any given pathway performed in different cell types.

### Assay timing

**Figure 5:**
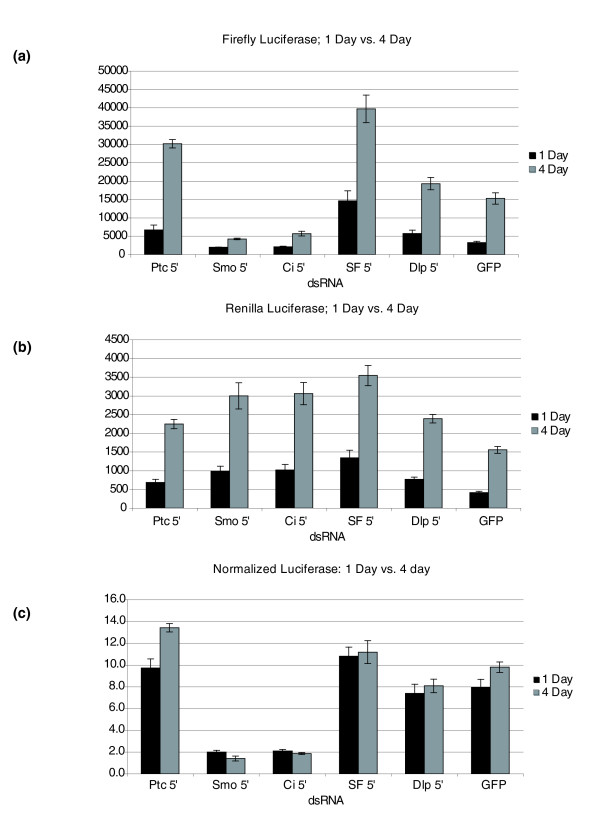
Hh assay timing. Hh signaling assays were conducted on identical assays plates with luciferase assays being scored after one or four days. **(a) **Comparison of RL counts when luciferase activity was assayed after one or four days in the presence of the indicated co-transfected dsRNA. **(b) **Comparison of firefly luciferase counts when luciferase activity was assayed after one or four days in the presence of the indicated co-transfected dsRNA. **(c) **Normalized luciferase values derived from the data in (a) and (b) of one-versus four-day assays. All luciferase reporter assays were performed in triplicate and error bars represent the standard error between the three data points.

Another variable that can affect transfection-based luciferase assays is the interval between reporter transfection and the luciferase assay. This interval can be important in allowing time for protein expression/accumulation, recovery from transfection, and post-translational modifications. It has recently been suggested that the interval between reporter gene transfection and luciferase assay may affect reporter activity in Hh luciferase based assays. Specifically, Ma *et al*. [27] have found that longer incubation periods after transfection reduces the fold difference in luciferase activity between the Hh stimulated and unstimulated states of their conditioned media-based Hh assays. In our initial characterization of the Hh assay, we had examined this possibility by how a one-day versus a four-day interval between transfection and luciferase assay might affect the Hh assay in clone 8 cells. As expected, we found that conducting the luciferase assays one day after transfection gave firefly luciferase values considerably lower than those obtained when the assays were conducted four days after transfection (Figure 5a). RL values were similarly lower in the one day assay compared to the four day assay (Figure 5b), and, in most cases, were near 10× background, which tends to average between 50 and 100 counts in cells not transfected with any reporters (KN, data not shown). Interestingly, the normalized values were very similar for the one day and four day assays, indicating that the interval between transfection and luciferase assay is not a strong modulator of Hh reporter activity (Figure 5c). However, since we find that it is best to keep control reporter activity well above ten times background levels, we opted for a greater than four day interval between transfection and luciferase assay. The strong effect that Ma *et al*. saw in their assay is likely due to the fact that they used conditioned media containing the artificially truncated form of Hh, Hh-N, as the source of Hh stimulus. This Hh-N source is likely highly labile in addition to being of undetermined Hh activity, and probably accounts for the reduction in fold difference of their Hh assay with longer incubation periods.

## Conclusion

RNAi technology has great potential to advance the field of signal transduction and cancer biology since it provides a direct method to systematically identify genes involved in signaling pathways implicated in development and disease. However, as with the development and application of most new and fast evolving technologies, a number of issues associated with rates of false positives and negatives have emerged in RNAi HTSs. In light of our experience and the lessons learned about the technology in recent years, we have examined the reproducibility of the Wg and Hh transcriptional reporter HTSs performed in our laboratory [3,6].

### Reproducibility of data from RNAi screens

As OTEs associated with long dsRNAs had been recognized to be a source of false positives in RNAi screens [26,27], we re-screened the majority of the 'hits' identified in the previously reported Wnt/Wg and Hh screens using independent validation dsRNAs that were free of predicted off-targets (based on a 19 nt sequence identity criterion). Our analyses revealed that a majority of candidate genes (from 51-73%) identified in the Wg and Hh screens could be re-validated using at least one independent dsRNA. Importantly, 58% of the dsRNAs in the Wg screen that were predicted to have OTs, and potentially be a source of false positives, could be confirmed with validation dsRNAs, suggesting that the mere detection of 19 nt homologies from computational analyses leads to an overestimation of the prevalence of OTEs. However, we do confirm that it is predictive, as a significantly higher proportion (85%) of our original DRSC1.0 dsRNAs with ≥5 19 nt cross-hybridizing sequences could be confirmed with the new dsRNAs in the Wg signaling assay, and 64% could be confirmed in the Hh assay.

Surprisingly, validation dsRNAs used in this study failed to identify some known negative regulators of Wnt signaling, such as *axn*, skpA and *slmb*, and some known negative regulators of Hh signaling, such as *ptc *and *slmb*, using either one or two independent dsRNAs. This underscores an important aspect of the validation process: although necessary, re-screening with new dsRNAs alone may not be sufficient in ruling out false positives in any specific screen. Since it may be non-trivial to design two or three independent OT-free dsRNAs that are comparable in their efficiency/ability to knockdown a target gene, screeners need to consider a balance between expunging the false positives and increasing the false negative rates in HTSs. Undoubtedly, the ultimate test for the validity of the candidate genes identified in any RNAi HTS lies in the validation of their function *in vivo *using traditional genetic and biochemical approaches.

### Reproducibility with screens from other laboratories

In light of the inherent noise associated with RNAi HTSs, it is of interest to compare the differences between similar studies and attempt to understand the sources of discrepancy. In the screen published for the Wg pathway in S2R+ cells by Ma *et al*. [27], the authors point to disparities between the results of their RNAi screen and the one performed in clone 8 cells in our laboratory. The major reason for this disparity was ascribed to the prevalence of OTEs caused by tandem trinucleotide 'CAN-repeats' that were present in some of the long dsRNAs of the DRSC1.0 library. Importantly, some of the candidate dsRNAs that were reported by DasGupta *et al*. [3] were shown to share short sequence homology with the *arm *gene, a critical regulator of the Wg pathway. While some of the differences can be explained by OTEs, other factors need also to be considered to account for the differences between the screens, including cell-type specific differences and differences in assay design. These include the use of different Wg-responsive reporters and control Renilla vectors, as well as differences in plate formats, protocols and assay conditions. Importantly, while the introduction of high concentrations of 30-40 bp short dsRNAs sharing sequence homology with multiple genes can clearly result in OTEs, it is difficult to predict, first, whether these siRNAs are even created in the cell upon the introduction of long dsRNAs, and second, when such an siRNA is created, whether its individual concentration in the siRNA pool would be sufficient to cause OTEs.

The initial screen for the Wg pathway also identified several genes that had been previously reported in genetic screens designed to find genes that could interact with the Wg pathway, including *lilli*, *brahma*, *osa*, *cdc2*, *string *(cdc-25), *N*, *mastermind *(*mam*), and so on. Although some of these dsRNAs have predicted OT sequences, this prediction alone should not necessarily deter any effort to follow them up, nor should it *a priori *negate their validity as true interactors. A more fruitful exercise would be to compare multiple forward- and reverse-genetic screens and protein-interaction screens in order to judge the validity of candidate genes in one specific screen.

It is also encouraging to find independent reports of new regulators being discovered for the Wnt pathway in different model systems that were also identified in our Wg screen with the DRSC1.0 library [3]. For example, a recent report described the function of P68 RNA helicase, an ortholog of the *Drosophila *Rm62 gene identified in the Wg-sreen. This protein was described to cause the dissociation of Axn from β-catenin and promote the nuclear translocation of the latter, thereby causing epithelial to mesenchymal transformation in human colon cancer cell lines [38]. Additionally, we isolated the *Drosophila *Tip60/CG6121, which had not been identified in prior genetic screens for the Wg pathway. However, recent studies in human cells and colorectal cancer cell lines have shown that the β-catenin carboxy-terminal activation domain associates with TIP60/TRAPPP and a mixed-lineage-leukemia (MLL1/MLL2) SET1-type chromatin-modifying complex *in vitro*, and that this complex promotes H3K4 trimethylation at the c-Myc target gene *in vivo *[39-41]. Similarly, in the Hh screen, the *roadkill *(*rdx*) gene (CG9924), encoding a ubiquitin ligase component, was identified as a negative regulator of Hh signaling in our screen. It was subsequently identified as a regulator of Hh signaling using traditional genetics means [42,43].

In conclusion, it is important to recognize that whole-genome RNAi screens using cell-based assays provide a technology platform for efficient enrichment for potential modulators of cell signaling pathways. Undoubtedly the ultimate validation will be in determining the function of the candidate genes *in vivo *in animal model systems, which is underway for several candidate genes obtained in the Wg and Hh screens (R DasGupta, RT Moon, and K Nybakken, unpublished). Additionally, our current understanding of OTEs associated with long dsRNAs is likely to be incomplete, and there may be other predictors (for example seed regions [22]) that, under the given circumstances, need to be avoided. Clearly our efforts in designing better reagents are still evolving and they will continue to be a major focus of further investigation. The early experience with RNAi reagents has led to a better understanding of their specificities and has already resulted in useful recommendations for best usage of the technology. With this and future knowledge in hand, we expect to see many exciting applications in the next few years of this powerful technology. (Note: for further information about OTEs, please visit the *Drosophila *RNAi Screening Center [43].)

## Materials and methods

### Generation of validation dsRNAs

PCR products with T7 polymerase sites on both ends for production of validation dsRNAs were obtained from the DRSC. They were further amplified by PCR using T7 primers and Takara (Tokyo, Japan) Taq polymerase and buffers. dsRNA was then produced using the Megascript kit (Ambion, Austin, Texas, USA). For T7 transcription, 6 μl of the T7 PCR reaction was used in a 2× (40 μl) Megascript transcription reaction. dsRNAs were digested with DNAse for 30 minutes at 37°C, then purified using Multiscreen purification plates (Millipore, Billerica, Massachusetts, USA) according to the manufacturer's instructions. dsRNAs were then quantified by spectrophotometry and diluted to 15 ng/μl in deep-well 96-well storage plates. Validation screening plates were then generated by arraying dsRNAs from 4 × 96-well storage plates into 384 well screening plates. For the screening plates, 75-100 ng of experimental or control dsRNA in 5 μl water was aliquoted per well, the plates sealed, and then frozen at -20°C until use.

### Wg screen

We assayed a minimum of three replica plates for each DRSC-v dsRNA, the average of which is reported in this study. A 30% increase or decrease in reporter activity with respect to GFP dsRNA control was considered significant, based on the effect of DRSC-v dsRNAs directed against known regulators of the Wg pathway, such as *wg*, *dsh *and *fz*. The log ratio of normalized luciferase units were computed as log(N-drsc_v/N-gfp) and plotted on the bar graph (in Additional data file 4). Luciferase reporter assays were performed using protocols previously described in [3]. All luciferase assays were performed in 96-well plate format using 25 ng each of Wg reporter and control Renilla vectors and 50 ng of inducer cDNA (pAct-wg). Cells were incubated with 100 ng dsRNAs for 4.5 days and luminescence measured using the EnVision plate reader (Perkin Elmer Life Sciences Inc., Waltham, Massachusetts, USA).

### Hh screen

Hh assays were conducted as previously described [6]. The validation assays were conducted three times. Normalized luciferase scores were converted to percentage changes with respect to GFP dsRNAs included in the plates as internal controls. These percentage changes were then averaged to give a final percentage change.

### Timing and Renilla control reporter assays

Assays were conducted as per [6] in 384-well plates, but 25 ng instead of 15 ng of the indicated Renilla control reporter were transfected and four replicate wells were assayed for each control reporter. Firefly and RL assays were conducted at the indicated times.

### Western blotting

Standard protocols were used for cell lysis, PAGE and western blotting. The anti-Axin antibody was used at 1:1,000 dilution in 5% milk in TBST (0.1% tween) buffer at 4°C overnight (O/N). HRP-conjugated secondary antibodies were used at 1:1,000 for 2 h at room temperature and the Pierce Supersignal WestPico Chemiluminescent kit (Pierce Biotechnology Inc., Rockford, Illinois, USA was used for detection.

## Abbreviations

Act-RL, Actin5C-RL; DRSC-v, DRSC-validation; dsRNA, double-stranded RNA; GFP, green fluorescent protein; Hh, Hedgehog; HTS, high-throughput screens; miRNA, microRNA; nt, nucleotides; OT, off-target sequence; OTE, off-target effect; RLU, relative luciferase units; RL, Renilla luciferase;  RNAi, RNA interference; siRNA, small interfering RNA; Wg, Wnt/Wingless.

## Authors' contributions

RD was responsible for research design, assays, data collection and analysis for Wg signaling and for manuscript production. KN was responsible for assays, data collection and analysis for Hh signaling as well as manuscript production. MB provided computational analyses for design and generation of validation dsRNAs. BM-P designed and generated validation dsRNAs. FG and BC were responsible for assays and data collection.  NP was responsible for research design, generation of validation dsRNAs and manuscript production. 

## Additional data files

The following additional data are available with the online version of this paper.

Additional data file 1 is a table listing the gene name, Curated Genes in the Drosophila genome based on gene predictions and previously characterized genes (CG#), and DRSC amplicon ID for all the new dsRNAs belonging to the DRSC-v library. The log-ratio of normalized luciferase units of experimental dsRNA (Nexp) with that of GFP dsRNA (Ngfp) is listed. Experiments were performed twice in triplicates (six data points for each gene tested). A consistent increase or decrease of at least 30% of the reporter activity with respect to the average of multiple negative controls (GFP dsRNA) was considered as a positive hit. Validation information for a second dsRNA is also provided for the genes that could be validated by the first amplicon. Additional data file 2 is a table listing genes name and CG# provided for those dsRNAs that were reported to have multiple potential OTs in the previously published Wnt/wg screen of DasGupta *et al*. [3], but still pass the validation test with DRSV-v dsRNAs (first column). Also listed are genes/CG# representing dsRNAs that represent unique amplicons in the DasGupta *et al*. screen and still pass with validation dsRNAs of the DRSC-v library (column 2). Note that several dsRNAs of the DRSC1.0 library that were thought to have OTEs could be re-validated using unique DRSC-v amplicons. Moreover, not all unique dsRNAs of the DRSC1.0 library had reproducible effects on the modulation of the Wg reporter activity when a corresponding unique validation dsRNAs (DRSC-v) was used. Additional data file 3 is a table listing the gene name, CG#, and DRSC amplicon number for all of the new dsRNAs tested in the Hh luciferase reporter assay. The number of potential off-targets calculated for the amplicon that was identified in the original Hh screen, based on a 19 bp window, is listed once for each gene. The average fractional change in reporter activity compared to GFP dsRNA controls (listed at the bottom) are presented, with scores between -0.25 and -0.50 highlighted in yellow, scores less than -0.50 highlighted in orange, and scores greater than or equal to + 0.50 highlighted in blue. At the bottom of the list, scores for GFP, Ci, Smo, and th dsRNA controls that were included in the assay plates are also indicated.

## Supplementary Material

Additional data file 1 The log-ratio of normalized luciferase units of experimental dsRNA (Nexp) with that of GFP dsRNA (Ngfp) is listed. Experiments were performed twice in triplicates (six data points for each gene tested). A consistent increase or decrease of at least 30% of the reporter activity with respect to the average of multiple negative controls (GFP dsRNA) was considered as a positive hit. Validation information for a second dsRNA is also provided for the genes that could be validated by the first amplicon. Click here for file

Additional data file 2 Genes name and CG# provided for those dsRNAs that were reported to have multiple potential OTs in the previously published Wnt/wg screen of DasGupta *et al*. [3], but still pass the validation test with DRSV-v dsRNAs (first column). Also listed are genes/CG# representing dsRNAs that represent unique amplicons in the DasGupta *et al*. screen and still pass with validation dsRNAs of the DRSC-v library (column 2). Note that several dsRNAs of the DRSC1.0 library that were thought to have OTEs could be re-validated using unique DRSC-v amplicons. Moreover, not all unique dsRNAs of the DRSC1.0 library had reproducible effects on the modulation of the Wg reporter activity when a corresponding unique validation dsRNAs (DRSC-v) was used. Click here for file

Additional data file 3 The number of potential off-targets calculated for the amplicon that was identified in the original Hh screen, based on a 19 bp window, is listed once for each gene. The average fractional change in reporter activity compared to GFP dsRNA controls (listed at the bottom) are presented, with scores between -0.25 and -0.50 highlighted in yellow, scores less than -0.50 highlighted in orange, and scores greater than or equal to + 0.50 highlighted in blue. At the bottom of the list, scores for GFP, Ci, Smo, and th dsRNA controls that were included in the assay plates are also indicated. Click here for file

Additional data file 4 *Drosophila *Cl8 cells were transfected with validation dsRNAs and the Wg-responsive luciferase reporter (dTF12). The log ratio of normalized luciferase units were computed as log(N-drsc_v/N-gfp) and plotted on a bar graph. Candidate negative and positive regulators are represented by negative and positive log ratios respectively, as compared to the GFP dsRNA control. Since the ratio of N_gfp/N_gfp is 1, the log ratio for gfp dsRNA control is zero. Click here for file
