# Supplementary figures and images for: A case study of the reproducibility of transcriptional reporter cell-based RNAi screens in Drosophila
Source: Genome Biol. 2007 Sep 28;8(9):R203. doi: 10.1186/gb-2007-8-9-r203 (PMC2375041; doi:10.1186/gb-2007-8-9-r203)

## Slide 1
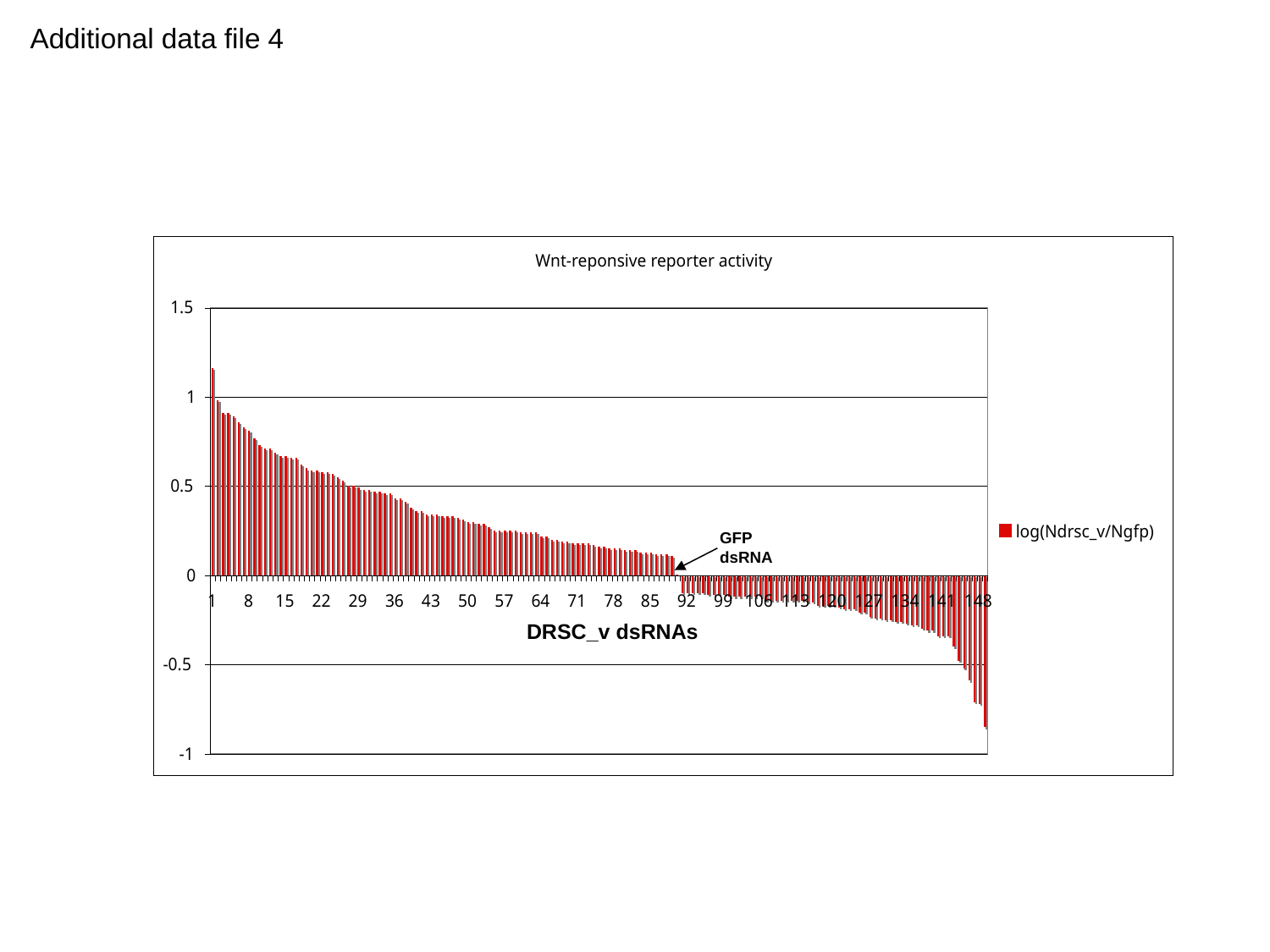

Additional data file 4
GFP
dsRNA
DRSC_v dsRNAs

Supplement: Additional data file 4 — Drosophila Cl8 cells were transfected with validation dsRNAs and the Wg-responsive luciferase reporter (dTF12). The log ratio of normalized luciferase units were computed as log(N-drsc_v/N-gfp) and plotted on a bar graph. Candidate negative and positive regulators are represented by negative and positive log ratios respectively, as compared to the GFP dsRNA control. Since the ratio of N_gfp/N_gfp is 1, the log ratio for gfp dsRNA control is zero. [file gb-2007-8-9-r203-S4.ppt]
